# The Stromal and Immune Landscape of Nasopharyngeal Carcinoma and Its Implications for Precision Medicine Targeting the Tumor Microenvironment

**DOI:** 10.3389/fonc.2021.744889

**Published:** 2021-09-10

**Authors:** Lanqi Gong, Dora Lai-Wan Kwong, Wei Dai, Pingan Wu, Yan Wang, Anne Wing-Mui Lee, Xin-Yuan Guan

**Affiliations:** ^1^Department of Clinical Oncology, Li Ka Shing Faculty of Medicine, The University of Hong Kong, Hong Kong, Hong Kong, SAR China; ^2^Department of Clinical Oncology, The University of Hong Kong-Shenzhen Hospital, Shenzhen, China; ^3^Department of Surgery, The University of Hong Kong-Shenzhen Hospital, Shenzhen, China; ^4^Department of Pathology, The University of Hong Kong-Shenzhen Hospital, Shenzhen, China

**Keywords:** nasophanrygeal carcinoma, tumor microenvionment, precision medicine, single-cell sequencing, immune regulation

## Abstract

The evolution of the tumor microenvironment (TME) is a cancer-dependent and dynamic process. The TME is often a complex ecosystem with immunosuppressive and tumor-promoting functions. Conventional chemotherapy and radiotherapy, primarily focus on inducing tumor apoptosis and hijacking tumor growth, whereas the tumor-protective microenvironment cannot be altered or destructed. Thus, tumor cells can quickly escape from extraneous attack and develop therapeutic resistance, eventually leading to treatment failure. As an Epstein Barr virus (EBV)-associated malignancy, nasopharyngeal carcinoma (NPC) is frequently infiltrated with varied stromal cells, making its microenvironment a highly heterogeneous and suppressive harbor protecting tumor cells from drug penetration, immune attack, and facilitating tumor development. In the last decade, targeted therapy and immunotherapy have emerged as promising options to treat advanced, metastatic, recurrent, and resistant NPC, but lack of understanding of the TME had hindered the therapeutic development and optimization. Single-cell sequencing of NPC-infiltrating cells has recently deciphered stromal composition and functional dynamics in the TME and non-malignant counterpart. In this review, we aim to depict the stromal landscape of NPC in detail based on recent advances, and propose various microenvironment-based approaches for precision therapy.

## Introduction

**Figure 1:**
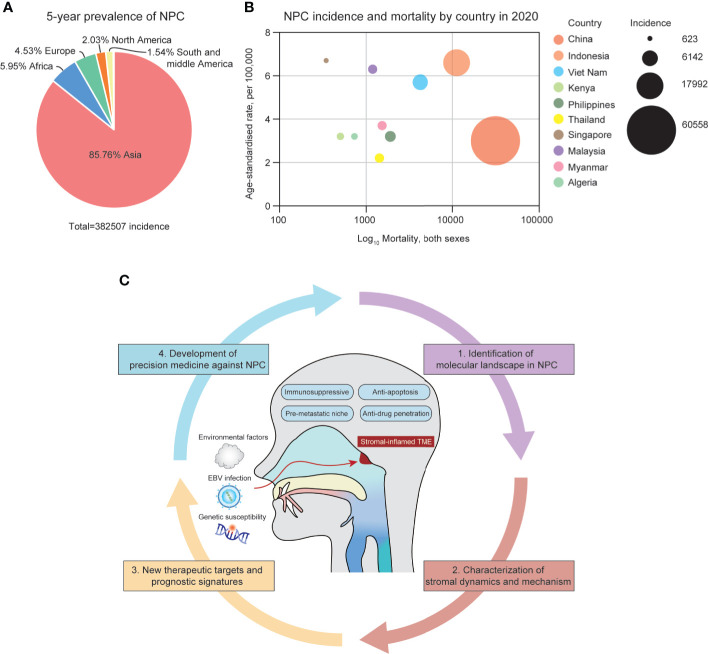
**(A)** The 5-year prevalence of NPC incidences around the globe from 2015-2020. **(B)** The geographical distribution of NPC incidences in Asia, 2020. **(C)** The summary of the NPC microenvironment and focuses of the review.

NPC is a unique type of cancer in terms of its geographical distribution, differentiation grade and microenvironmental landscape. According to the global cancer statistics in 2020, more than 75% of NPC cases were diagnosed in East and Southeast Asia, especially in southern China (1) (**Figures 1A, B**). People who have ancestors who originally resided in southern China possess a higher possibility of NPC incidences, indicating that NPC pathogenesis might be closely related to epidemiological patterns and genetic susceptibility in certain ethical groups (2). In addition, undifferentiated NPC (The World Health Organization type III histology) is the predominant disease type that constitutes more than 90% of total incidences, in which tumor cells exhibit many stem-cell-like signatures, including CD133, CD44 and ALDH1 (3–6). The NPC microenvironment might provide a supportive niche for such a high portion of undifferentiated cells. Compared with many solid tumors, NPC has the most severe stromal infiltration, possibly because NPC is originated from the nasopharynx that contains secondary/tertiary lymphoid structures (TLSs) and closely associated with EBV infection (7). Even EBV-negative NPC tumors and non-malignant nasopharyngeal tissues are also intensively infiltrated with varied stromal cells, caused by the locoregional lymphoid structures and tumor-mediated mechanism (8, 9). However, the differentiated NPC is significantly less infiltrated with stromal cells, suggesting that pathological status might alter cellular composition in the NPC microenvironment. During the past decades, the tumor heterogeneity and stromal landscape in the NPC microenvironment remain largely unexplored. Only few studies have reported that T cell and myeloid-derived cells are the predominant stromal subtypes in the NPC microenvironment based on hematoxylin and eosin (H&E) staining, immunohistological (IHC) staining and flow cytometry (10–12). Other stromal cells, such as fibroblasts and B cells, have not been comprehensively characterized in the NPC microenvironment yet, but might be associated with stemness, therapeutic resistance, and immune regulation (13, 14). However, these techniques using few gene signatures remain far from sufficient to identify the finer stromal subpopulations and characterize the functional dynamics of those tumor-infiltrating cells on immune suppression and tumor progression.

The lack of understanding of the stromal landscape in NPC significantly hinders the development of precision medicine. Conventional chemotherapy (cisplatin, gemcitabine and fluorouracil) and radiotherapy have been facing obstacles in optimizing the efficacy in locoregional advanced NPC, overcoming acquired resistance, and suffering from long-term toxicities (15–17). Recently, immunotherapy has emerged as a new strategy to treat recurrent, metastatic, and chemo/radio-resistant NPC patients using PD-1 inhibitors, including camrelizumab, pembrolizumab and nivolumab (18–20). Although the PD-1-based therapeutics has been shown effective in phase I/II clinical trials, it has also suffered from patient-specific responsiveness and adaptive resistance after long-term dosage. Resistance to immunotherapy is multifaceted since the functional state of infiltrating stromal cells is dynamic. Thus, in order to optimize precision medicine in NPC patients, it remains essential to comprehensively decipher the stromal landscape in NPC, and to identify patient-specific targets and signatures associated with prognosis and treatment responsiveness (**Figure 1C**).

Single-cell sequencing has provided a powerful platform to analyze the heterogeneous ecosystem in cancer. As yet, research enthusiasm for single-cell sequencing has remained high, and the TME-infiltrating cells in many cancers have been revealed. Single-cell analysis has provided bench-to-bedside guidelines to clinical practice, especially by revealing TME-based targets that can re-activate immune response or inhibit tumor-facilitating effects. However, the single-cell sequencing of NPC only started after 2019, largely due to its low global incidence. So far, single-cell data of NPC have participated in the public repository and contributed to the establishment of large-scale and multi-central cohorts for downstream analysis (21). In this review, we aim to address the stromal landscape in NPC based on recent advances, and subsequently propose a variety of approaches to enhance therapeutic response and patient prognosis *via* specifically targeting the immunosuppressive and tumor-promoting microenvironment.

## The Heterogenous NPC Microenvironment Shaped by Locoregional Lymphoid Infiltration, EBV Infection and Tumor-Mediated Recruitment

NPC is categorized as an inflamed tumor based on its spatial localization of stromal cells with respect to tumor compartments (22). Stromal cells are in close proximity to and in contact with NPC cells, instead of being embedded in the surrounding regions away from the tumor core. Hence, cytokine secretion and ligand-receptor interactions are both involved in the bilateral tumor-stroma interplay. It is also noteworthy that the nasopharynx is one of the first defensive organs against viral and bacterial entry and infection, which makes its underlying microenvironment highly heterogenous and immunogenic prior to malignant transformation.

There exist two major cell lineages in the nasopharyngeal microenvironment, CD45^+^ immune cells, including T cells, B cells, natural killer cells (NK cells), and myeloid-derived cells, as well as CD45^-^ non-immune stromal cells, including fibroblasts and endothelial cells. Recent single-cell analysis has revealed that normal nasopharyngeal tissues also have high immune infiltration, especially for T and B lymphocytes (23, 24). Fibroblasts and myeloid-derived cells are hardly seen from normal nasopharyngeal tissues, even those with reactive hyperplasia caused by allergy and inflammation (23, 24). The major stromal landscape between normal nasopharyngeal tissues and malignant nasopharyngeal carcinoma is distinctive. For instance, B cells are highly enriched in the normal nasopharyngeal tissues upon inflammation, whereas T cells, NK cells, myeloid-derived cells and fibroblasts, are more likely to infiltrate the NPC microenvironment (23). Germinal centers are commonly seen in the normal nasopharyngeal tissues, where CD3^+^/CCR7^+^ naïve T cells and CD19^+^/CD27^-^ naïve B cells accumulate and proliferate, causing lamps in the nasopharynx (23, 25). Under non-malignant inflammation, a large portion of those naïve lymphocytes does not differentiate into cytotoxic, memory and regulatory phenotypes. However, the chronic EBV infection and tumor progression result in an increasing number of naïve cells transitioned into an activated state, and eventually become exhausted (26). Retrospective cohort studies have found that 90% of NPC incidences are accompanied by EBV infection, but there still are EBV^-^ cases where the stromal composition is distinctive from EBV^+^ counterparts (27). The abundance of major cell lineages in EBV^-^ NPC patients, does not significantly differ from the abundance in the EBV^+^ microenvironment, but the exhausted and immunoregulatory subtypes, such as HAVCR2^+^/PD-1^+^ T cells, CD25^+^/FOXP3^+^/CTLA4^+^ regulatory T cells (Tregs) and CD68^+^ myeloid-derived cells are found more enriched in the EBV^+^ microenvironment (11, 28). Besides, the functional state of infiltrating immune cells has been greatly influenced by the hyper-activation of interferon (IFN) secretion induced by EBV infection. In the NPC microenvironment, type I and type II IFNs, namely IFN-α and IFN-γ, are activated to combat viral entry and incorporation. Thus, the IFN-α and IFN-γ signaling pathways are activated in almost all the infiltrating immune cells, mainly reflecting in up-regulation of IFN-induced genes, including ISG15, IFI6, IFI44L, IFIT3 and IFITM1 (23). On the contrary, NF-κB signaling is up-regulated in nasopharyngeal tissues upon non-malignant inflammation, but it is also closely associated with inflammation caused by EBV-encoded genes (23). Although IFNs play a vital role in anti-tumor cytotoxicity, previous studies have reported that chronic IFN activation in the TME hijacks helper T cell response and leads to progressive exhaustion of T cells and persistent infection (29–31). Nevertheless, the molecular mechanism of chronic IFN activation on immune cells needs to be further investigated, including its effect on antigen presentation, T cell differentiation, activation and exhaustion.

EBV infection in NPC is classified as type II latency and contributes to TME remodeling. Type II latency is characterized by the expression of a set of latent genes in NPC, including Epstein-Barr nuclear antigen 1 (EBNA1), latent membrane protein 1 (LMP1), LMP2 and Epstein-Barr encoding region (EBER) RNAs (32, 33). LMP1 and EBNA1 expressed by NPC cells are able to induce PD-L1 up-regulation *via* STAT3 and NF-κB signaling, Treg recruitment *via* CXCL12-CXCR4 chemotaxis, as well as expansion of myeloid-derived suppressor cells (MDSCs) (31, 34, 35). EBV can also infect B cells, however, the infiltrating B cells in the NPC microenvironment are uninfected, indicating that EBV infection on nasopharyngeal epithelial cells may occur prior to B-cell recruitment and accumulation (36). Tumor-mediated recruitment is another mechanism to alter the microenvironmental landscape. NPC cells specifically express cytokine-encoding genes, including CX3CL1, CXCL10, CCL2, CSF1, IL-10 and TGF-β1 that are critical for the recruitment of immune cells from peripheral blood and immune suppression (37, 38). T-cell receptor (TCR) profiling has revealed that CX3CR1^+^ T cells are migrated from the peripheral blood into the NPC microenvironment *via* CX3CL1-CX3CR1 chemotaxis. Differentiated NPC has higher macrophage infiltration, lower B cell infiltration and worse prognosis compared with undifferentiated NPC (39, 40). Although Longitudinal analysis has suggested that the peripheral myeloid-to-lymphocyte ratio negatively correlated with overall survival in NPC patients, little is known whether the prognosis is directly influenced by the abundance of macrophages and B cells in the TME (41).

Other head and neck squamous cell carcinoma (HNSCC) developed from the oral cavity, oropharynx, hypopharynx, and larynx, also displays an inflamed microenvironmental landscape due to NF-κB activation and immune evasion (42, 43). Human papillomavirus (HPV), instead of EBV, plays a vital role in the immune modulation of HNSCC. HPV^+^ HNSCC is infiltrated with a higher number of Tregs, CD20^+^ B cells, and NK cells, but a lower number of T helper cells than its HPV^-^ counterpart and non-malignant inflamed tonsil (42, 44). HPV infection also causes T cell dysfunction, possibly *via* the overreaction of IFN-associated signalings and HPV integration into the host genome (42, 45). B cell infiltration in HPV-associated HNSCC is as high as in NPC and similarly correlates to a better prognosis (44). Germinal centers and TLSs are frequently seen in HPV^+^ tumors, associated with better patient survival and responsiveness to ICB therapies (44, 46). PD-1/PD-L1 blockades combined with chemotherapeutic drugs such as platinum and fluorouracil have prolonged the overall survival by three months in patients with advanced and metastatic HNSCC (47). However, the objective response rates of nivolumab and pembrolizumab in HNSCC patients are only 15%, significantly lower than in NPC patients (48, 49). The paradigm-shifting therapeutics in NPC, including alleviating viral infection, inactivating Treg-mediated suppression and expanding TLS-associated B cells within the TME, might also be feasible in HPV^+^ HNSCC to synergistically promote PD-1/PD-L1 efficacy.

The severe stromal infiltration in the NPC microenvironment is not solely shaped by one factor, but a combination of factors that lead to the phenomenon we have seen in clinical practice. Amidst the complexity of the NPC microenvironment, many therapeutic targets remain effective to modulate the tumor-stroma interplay, which imparts strong influences on tumor progression and therapeutic resistance and responsiveness. For instance, while exhausted and regulatory T and myeloid-derived cells exhibit immunosuppressive function in response to cytokine stimulation and antigen presentation in NPC, they can also be reprogrammed to reinvigorate tumor-specific cytotoxicity *via* pharmacological administration. The plasticity of the NPC microenvironment has offered an approach to specifically re-activate dysfunctional subtypes and in-activate suppressive subtypes to achieve optimal anti-tumor effects, which requires an in-depth understanding of the stromal phenotyping and functional dynamics.

## Targeting T Cells and NK Cells to Reinvigorate Tumor-Specific Immunity

Tumor survival from the host immune system is one of the critical steps during malignant progression, which can be achieved *via* inhibition of cytotoxic cells and activation of immunosuppressive cells. Naïve T cells are intrinsically enriched around the germinal centers in the nasopharynx. As a consequence of tumor progression, the normal T-cell differentiation and activation processes can be hijacked by NPC cells and eventually result in the dominance of dysfunctional and suppressive T cells in the NPC microenvironment. The inhibitory signatures on CD8^+^ T cells, including PD-1, HAVCR2 and LAG3 have been found up-regulated by EpCAM^+^HLA-DR^High^ NPC cells *via* ligand-receptor interaction (50). TCR profiling on NPC-derived T cells has validated the presence of activation-to-exhaustion transition, where a portion of activated effector T cells gradually loses its cytotoxic function (23). Indeed, the exhaustion program is dynamic instead of terminally static. Most of the exhausted T cells can still secrete cytotoxic cytokines, particularly IFN-γ and granzymes (GZMs), but at a lower level than fully activated effector cells. Therefore, a high abundance of exhausted and activated T cells usually correlated to better prognosis and higher immunotherapeutic responsiveness in NPC patients (23, 24).

Targeting CD8^+^ T cells primarily focuses on how to inhibit and reverse the activation-to-exhaustion transition. Targeting inhibitory checkpoint molecules has been shown effective in the context of anti-tumor immunity, in which PD-1 is currently the only therapeutic target for CD8^+^ T cells in NPC clinical trials (20, 51). The clinical response rate for PD-1 monotherapy using camrelizumab, nivolumab and pembrolizumab ranges from 20.5% to 34% in phase I/II clinical trials (18–20, 52). Although the response rate for pembrolizumab in NPC (phase I, 25.9%) is significantly higher than in non-small-cell lung cancer (phase I, 19.4%), hepatocellular carcinoma (phase III, 18.4%) and gastroesophageal cancer (phase II, 11.6%), synergistically targeting more highly expressed receptors in NPC-infiltrating exhausted T cells might enhance the anti-tumor immunity of PD-1 monotherapy (53–55). HAVCR2^High^ exhausted T cells had a unique exhaustion program but identical TCR clonotypes with PD-1^High^ counterparts, indicating that the exhaustion transition is not independent where exhausted T cells are maintained as a homogenous population with fixed molecular signatures (23). Conversely, T cells on an early, intermediate or late exhaustion stage, exhibited phase-specific inhibitory signatures and can be continually transformed from one stage to another upon stimulation. Single-cell sequencing and multiplex immunofluorescence has corroborated that HAVCR2, instead of PD-1, is the predominant inhibitory molecule in the NPC microenvironment (23, 24, 56). BGB-A425, as a humanized anti-Tim-3 (encoded by HAVCR2) antibody, is currently in progress of phase I/II clinical trials treating solid tumors in combination with tislelizumab, which has been shown to augments T-cell response *via* enhancing IFN-γ production and NK-mediated cytotoxicity (57). Galectin-9 (encoded by LGALS9), as the most studied ligand for HAVCR2, is specifically expressed by NPC cells, as an immunosuppressive molecule induced by high intratumoral IFN-β and IFN-γ (58, 59). Inhibition of LGALS9 selectively expands and activates infiltrating exhausted T cells by intervening in the crosstalk between PD-1 and HAVCR2 (59). Targeting IFN-induced LGALS9 up-regulation and secretion in NPC cells might be an alternative approach to overcome the primary and adaptive resistance to the PD-1/PD-L1 therapy.

LAG3 is another predominant inhibitory signature on infiltrating exhausted T cells in the NPC microenvironment (23, 24, 56). Unlike HAVCR2, LAG3 has been found specifically expressed on exhausted ZNF683^+^ tissue-resident T cells (23). The average abundance of tissue-resident memory T cells in the NPC microenvironment is approximately 10%, two-fold lower than the cytotoxic and exhausted T cells (23). It might be that a substantial amount of infiltrating cytotoxic T cells does not originally reside in the nasopharynx, but is recruited from peripheral blood. As previously stated, CD8^+^/CX3CR1^+^ T cells with minimal cytotoxicity and proliferative capacity are migrated from blood and quickly become exhausted *via* an EBV^+^ NPC-secreted cytokine, CX3CL1, constituting the major source of infiltrating CD8^+^ T cells in the TME (56). In phase I/II clinical trials, the efficacy of LAG3-targeted antibodies, such as MK-4280, TSR-033 and IMP321, are often evaluated in combination with anti-PD-1 and anti-HAVCR2 treatment to promote responsiveness (60, 61). Although therapeutic targeting to HAVCR2 and LAG3 is currently not as mature as to PD-1, we must pay attention that the NPC microenvironment is unique and complicated, which means that we cannot directly adapt a developed therapeutics from other malignancies into NPC treatment. In the future, the combo-therapy synergistically targets PD-1/HAVCR2 or PD-1/LAG3 might become a more effective therapeutic option for NPC patients.

Inactivation of Tregs is also an approach to retrieve immunosurveillance against NPC, which indirectly enhances anti-tumor T cell response. Similar to the exhausted subtypes in the NPC microenvironment, there exist two subtypes of Tregs, resting Tregs and suppressive Tregs, which both have high expression of Treg signatures, including CD25, FOXP3 and IKZF2 (23, 24, 56). The two immunoregulatory subtypes are functionally different since suppressive Tregs possess a higher expression of immune checkpoint CTLA4 and co-stimulatory molecules CD27, TNFRSF4, TNFRSF9 and ICOS (23, 24, 56). Anti-CTLA4 therapy using ipilimumab, has shown effective to improve overall survival in patients with melanoma and hepatocellular carcinoma, but is often used in combination with PD-1 inhibitors (62–65). The efficacy and safety of ipilimumab+nivolumab in NPC is currently under investigation in a phase II clinical trial (NCT03097939). Based on the preliminary data up to February 2020, the partial response rate was 35% with a median duration of response of 5.9 months, which is significantly higher than the responsiveness of PD-1-based monotherapy. The average abundance of CTLA4^+^ Tregs is approximately 20%, which might explain why NPC is responsive to anti-CTLA4 drugs that relieve Treg-mediated suppression and expedite proliferation of effector T cells (23). In addition, Treg-mediated suppression is largely dependent on CD27-CD70 interaction, which provides co-stimulatory signals critical for naïve-to-Treg differentiation, Treg proliferation and activation. Cusatuzumab (ARGX-110) is a CD70-targeting drug currently under clinical evaluation. Previous *in vitro* studies have demonstrated that blocking CD70^+^ leukemia and B cell lymphoma cell lines using ARGX-110 can inhibit the activation of Treg and facilitate the anti-tumor immunity exerted by CD8^+^ effector T cells (66). Most of the solid tumors lack CD70 expression, whereas only hematologic cancers have a high frequency of CD70^+^ cancer cells. Thus, cusatuzumab is mainly being evaluated in acute myeloid leukemia in phase II clinical trial (67). In the NPC microenvironment, CD70 is highly and specifically expressed on tumor cells rather than T cells and dendritic cells (DCs). The pathological examination has confirmed that more than 80% of NPC cases are CD70 positive (68). Therefore, direct targeting of CD70^+^ tumor cells might further inhibit Treg accumulation and activation in NPC. However, recent studies have suggested that CD70 deficiency in EBV-infected patients might exacerbate chronic EBV infection and predispose them to lymphoma and immune disorders (69, 70). As CD70 is also expressed on CD8^+^ effector T cells and activated B cells, lack of functional CD70 might hinder T and B cell-mediated immunity to combat EBV infection and further promote tumor progression. Thus, the safety of anti-CD70 therapy should be carefully examined in humanized animal models prior to clinical translation, for its potential to damage immunocompetence.

Accumulating evidence has suggested that the enrichment of Tregs in the NPC microenvironment is also caused by tumor-mediated recruitment, where CCR4^+^ and CCR6^+^ resting Treg are migrated from peripheral blood and activated into a suppressive phenotype *via* tumor-secreted CXCL10, CXCL16, CCL20, and CD70 binding (50, 71, 72). Targeting these Treg-attractive chemokines produced by NPC cells might also alleviate the infiltration of Tregs in the TME and sustain CD8^+^ cytotoxicity. Additionally, excessive production of IFNs might also contribute to Treg accumulation by hijacking the CD4^+^ naïve T cell differentiation, since CD4^+^ IFN-induced T cells in the NPC microenvironment co-express naïve signatures.

LGALS1 has been found highly expressed in suppressive Tregs in the NPC microenvironment and plays a vital role in Treg activation (23). Extracellular galectin-1 (encoded by LGALS1) has been implicated in promoting the suppressive capability of Treg and inducing apoptosis of CD8^+^ T cell (73). LGALS1-deficient mice showed impeded Treg activity. In T cell subpopulations, LGALS1 is correlated to CD25 expression, indicating it might play a vital role in Treg activation. Blocking and CRISPR-silencing of LGALS1 leads to decreased proliferation and IFN production in T cells (74). LGALS1 has long been considered as one of the key regulators in T-cell homeostasis and inflammation. However, its function in Treg has not been explicitly elucidated. LGALS1 up-regulation in Treg promotes growth arrest and apoptosis and inhibits the secretion of pro-inflammatory cytokines of activated T cells. Thus, it might serve as an immune checkpoint to revert the Treg-mediated suppression *via* partial activation. In HPV^+^ and HPV^-^ head and neck squamous cell carcinoma, LGALS1 blockade has resulted in elevated infiltration of T cells in tumor cores and further enhance response to PD-1 therapy (75). In clear cell renal carcinoma, patients who are responsive to PD-1 therapy possess a higher expression of LGALS1 (76). Thus, inhibition of LGALS1 might synergize with ICB-based monotherapy. TNF-alpha signaling and calcium channel might be regulated by LGALS1 to exert its effect on T cell survival and activation. LGALS1 has also been found expressed on NPC cells, further confirming that NPC cells are actively involved in immune regulation of the TME. Currently, there are no clinically available therapeutic agents that specifically target LGALS1, but targeting LGALS1 in NPC patients remains a feasible approach that worth to be developed in the future.

NK cells are commonly characterized by the high expression of GNLY, and they also express chemokines CCL5, XCL1 and XCL2 responsible for the recruitment of pro-inflammation CCR5^+^/XCR1^+^ DCs (77). Compared with cytotoxic T cell infiltration, NK cells constitute a relatively minor subpopulation (~2% of the total stromal infiltrates) (23). Previous studies have suggested the presence of dysfunctional NK cells with NKG2A, PD-1 and HAVCR2 up-regulation in the TME (78). In the NPC microenvironment, NK cells do not express these exhaustion signatures, instead, NK cells highly express cytotoxic signatures, indicating that NK cells might not be severely influenced by tumor cells and chronic infection (23). It offers a new perspective for anti-tumor immunity which we can expand or recruit immune-activated NK cells to counter the loss-of-function in exhausted T cells. Nevertheless, it remains necessary to evaluate the prognostic value of NK-specific signatures and investigate the mechanism so that we can have a better understanding of the role of NK cells in the NPC microenvironment that might facilitate therapeutic development in the future.

## Targeting B Cells to Enhance Response to Immunotherapy

Compared with T cells, significantly fewer B cells are often found in the TME (79). However, single-cell analysis in NPC has shown that B cells are more enriched and diverse than previously reported, and the infiltration and functionality of B cells have emerged as a vital prognostic factor and therapeutic target (23, 24, 56). Increased B cell density in the TME facilitates the establishment of TLSs and promotes responsiveness to PD-1/CTLA4 immunotherapy in melanoma (80, 81). The spatial localization and cell-cell communication have been observed in tumor-associated TLSs, where T cells and B cells can undergo cooperative maturation, activation and clonal expansion. In addition, a higher expression of B cell-associated signatures, including CD79A, CD20, CD27, IGHD, CXCR5 and FCLR4, are associated with increased progression-free survival in NPC patients (23). Enrichment of B cells might be caused *via* CXCL13-CXCR5 chemotaxis from surrounding lymph nodes and peripheral blood into the TME. In the NPC microenvironment, CXCL13 is mainly produced by CD4^+^ helper T cells and PD-1^+^ exhausted T cells, suggesting that exhausted T cells might remain beneficial to the immune modulation *via* the recruitment of CXCR5^+^ B cells (only plasma B cells are CXCR5^-^) and TLS development (23). In non-small-cell lung cancer patients who received PD-1 blockade, increased CXCL13 production also has been found in PD-1^+^ exhausted T cells with impaired cytotoxicity (82). Considering the positive prognostic value of tumor-infiltrating B cells, it might be an effective adjuvant therapy to increase intratumoral B cells *via* CXCL13-dependent recruitment in NPC patients with low TLS density. Consequently, TLSs provide a harbor for lymphocyte maturation and immune activation. Meanwhile, the molecular function and mechanism of infiltrating B cells and tumor-associated TLSs remain undiscovered owing to relatively few B infiltrates in most malignancies. Thus, NPC can serve as an applicable model to investigate the interplay within T cells, B cells and tumor cells.

A higher abundance of intratumoral B cells is frequently associated with better prognosis in NPC patients, but there exist B subtypes that contribute to worse prognosis (23, 24). Single-cell sequencing of NPC patients and non-malignant counterparts has identified the presence of double-negative B cells (IGHD-/CD27-) in the TME. Double-negative B cells are commonly found in the peripheral blood of patients with autoimmune diseases, such as rheumatoid arthritis and systemic lupus erythematosus (83–85). Although double-negative B cells represent a rare subpopulation in the normal microenvironment, they have been found expanded in the NPC microenvironment and constituted 12.6% of the total CD79A^+^ B cells, and correlated to worse prognosis (23). Increased double-negative B cells have also been found in non-small cell lung cancer and negatively correlated to the abundance of pro-inflammation B cells (86). Higher frequencies of double-negative B cells are associated with lymph node and distant metastasis in cancer patients, which might be alleviated or overcome by cisplatin-based chemotherapy (87). Nonetheless, little is known about the function nor the mechanism of double-negative B cell enrichment in the NPC microenvironment because they have not been previously detected in tumor tissues. One recent study has exhibited that double-negative B cells might regulate inflammatory activation and undergo clonal expansion upon antigenic stimulation *via* an extra-follicular maturation pathway (88). Pseudotime trajectory analysis has shown that double-negative B cells are the precursors of matured effector B cells, which can be further differentiated into plasma B cells and memory B cells (23). However, it seems that in the NPC microenvironment, the differentiation of double-negative B cells is hijacked by tumor- or TME-mediated mechanism so that a substantial portion of double-negative B cells are forced to maintain in an intermediately differentiated and ineffective phenotype. Hence, inhibiting the expansion or inducing the differentiation of double-negative B cells in the NPC microenvironment might enhance inflammatory activation. Furthermore, quantifying the intratumoral or peripheral abundance of double-negative B cells in NPC patients might be feasible for patient stratification and prognosis, as well as serve as a biomarker for treatment selection.

Antibody-secreting plasma B cells and FCLR4^+^ memory B cells represent two terminally differentiated pro-inflammation subtypes in the NPC microenvironment. They are commonly more infiltrated in the TME and correlated to better clinical outcomes, exhibiting their functions in immune survilliance (89). Nevertheless, their abundance is highly patient-specific due to EBV status, since plasma B cells and memory B cells showed increased enrichment and activity in EBV^+^ tumors than in EBV^-^ ones (23). The differentiation and activation of these two subtypes are influenced by chronic IFN-α and IFN-γ production and chemotaxis in the NPC microenvironment (23). In the treatment of autoimmune and infectious diseases, rituximab is used to deplete excessive enrichment of CD20^+^ B cells (90), whereas, in NPC treatment, we should focus on *in vivo* or *ex vivo* expanding pro-inflammatory B cells, especially in the NPC patients with fewer B infiltrates (91). Previous studies have illustrated that B cell proliferation and maturation can be re-directed *via* CD38-mediated inhibition of mTOR and PI3K signaling (92–94). However, the function of B cells and their associated antibody repositories in NPC is hardly identified and characterized so far. Thus, prior to clinical translation, it remains necessary to understand the crosstalk between plasma B cells/memory B cells and T cell subpopulations so that humoral immune responses stimulated by tumor-infiltrating B cells can facilitate the development of effective anti-tumor immunity within the TME.

## Targeting Myeloid-Derived Cells to Intervene the Tumor-Stroma Communications

Intratumoral myeloid-derived cells are commonly developed from immature monocytes recruited from peripheral blood during tumor progression and viral infection (95, 96). Immature macrophages and monocytes express pan-monocyte markers CD14 and CD68, but they lack functional and polarized signatures such as IL-10. Enrichment of these immature myeloid cells indicates an earlier or less progressive disease status, therefore its abundance is associated with better prognosis in undifferentiated NPC patients (97). Prevalent cell-cell communications have been identified between macrophage and lymphocytes, indicating the strong potential to recruit, activate and suppress innate and adaptive immunity. In the NPC microenvironment, the maturation and polarization of macrophages do not follow the classic M1/M2 model. Instead, the infiltrating macrophages exhibit an M1/M2 coupled pattern, expressing both M1 and M2-polarized signatures, including FCGR2A, FCGR3A, TREM2 and APOC1 (23). The multilateral cell-cell communications between macrophages and lymphocytes are ambiguous due to dynamic functional alterations. For example, CD163^+^ M2-polarized macrophages in the NPC microenvironment are considered to associate with worse prognosis and facilitate the development of a pre-metastasis niche by secreting pro-angiogenesis cytokines, including VEGF, MMP9, TGFB1 and PLA2G7 (97–99). Hypoxia and IFN signaling are two mechanisms that induce accumulation, maturation and M2 polarization of macrophages, and further induce therapeutic resistance and disease progression in the NPC microenvironment (100). Targeting molecules that are associated with hypoxia and IFN signaling represents a potential therapeutic strategy to minimize the suppressive function of M2 macrophages or enhance the accumulation of monocytes and M1-macrophages in the TME.

DCs are one of the differentiated myeloid subtypes accumulated both in nasopharyngeal hyperplasia and NPC, and responsible for antigen processing and presentation so that T and B cells can be activated (101, 102). For instance, FCER1A^+^ Langerhans cells and LGALS2^+^ DCs are tissue-resident DCs located in the epithelium and lymph nodes of the nasopharynx, to capture and recognize the antigens on malignant-transforming nasopharyngeal epithelial cells. Although DCs are a pro-inflammation subtype and significantly correlated to better prognosis in NPC patients, some DCs have been found capable of impairing T-cell and B-cell immunity driven by chronic inflammation and hypoxia (103). In the NPC microenvironment, LAMP3^+^ DCs with high maturation, immune-regulatory and migration potentials, produce multiple cytokines, including CCL17, CCL19 and CCL22, to recruit CCR4^+^ Tregs and CCR7^+^ naïve T cells (56). LAMP3^+^ DCs also exhibit reduced immune activation status and elevated suppression status, characterized by high expression of PD-L1, PD-L2, IDO1 and TGFB1. Meanwhile, the immunosuppressive function of LAMP3^+^ DCs has been validated in hepatocellular carcinoma (HCC) and non-small-cell-lung cancer (NSCLC) where LAMP3^+^ DCs showed strong interaction with exhausted T cells, Tregs and proliferating T cells *via* CD28/B7 binding and IL-15 signaling (104, 105). Up-regulation of LAMP3 in tumor tissues has been found correlated to worse prognosis in patients with esophageal squamous cell carcinoma (ESCC) (106). Developmental trajectory has revealed that LAMP3^+^ DCs might be differentiated from immature monocytes during tumor initiation so that NPC cells can escape from initial antigen recognition and immune attack (56). Thus, therapeutic targeting of LAMP3^+^ DCs might be only feasible in the early stage of NPC development so that effector lymphocytes can more effectively recognize the tumor antigens subsequently induce tumor depletion. However, the function of LAMP3 remains ambiguous since immunosuppressive DCs in metastatic lung adenocarcinoma have shown loss of LAMP3 activation marker expression (107). Although LAMP3^+^ DCs are common malignancy-associated infiltrates with the ability to restrain T cell function, further *in vivo* and *in vitro* functional assays need to be conducted to validate its regulatory potential.

MDSCs are another type of differentiated monocytes infiltrating in the TME (108). Unlike DCs which are infiltrated both in the malignant and non-malignant nasopharyngeal microenvironment, MDSCs are highly enriched in the NPC microenvironment, indicating the presence of NPC-associated recruitment and differentiation in MDSCs. Previous studies have considered that MDSCs are a dynamically changing myeloid subtype that cannot be accurately characterized *via* genetic profiling (103). Single-cell sequencing has demonstrated that infiltrating MDSCs are lack macrophage and DC-specific signatures, such as CD14 and CD68, but highly express S100A family genes, including FCN1, VCAN, S100A8 and S100A9 (105, 109). Accumulating evidence has suggested that S100A8/S100A9 are pro-inflammation molecules that are elevated in patients with a variety of inflammatory diseases and cancer (110, 111). The expression of S100A8/A9 is closely related to tumor stage, lymph node metastasis and poor prognosis in NPC patients (112). In tumor-bearing mice treated with mAbGB3.1 (10 ug/gm body weight), S100A8/A9 binding and signaling has been blocked and the accumulation of MDSCs in the peripheral blood and secondary lymphoid organs has been reduced (113). S100A8/A9^+^ MDSCs exert immunosuppressive effects *via* hijacking the differentiation from monocytes into antigen-presenting DCs and pro-inflammation macrophages *via* STAT3 signaling (114). Thus, limiting the accumulation and retention of MDSCs can facilitate the activation of anti-tumor immunity *via* communications with T and B cells, and might further retard NPC progression. Targeting S100A8 and S100A9 *via* siRNA has shown to reduce invasive capability of NPC cells, whereas the silencing effects on TME remodeling remain under-investigated (115).

## Targeting Fibroblasts to Manipulate the Tumor-Promoting Extracellular Matrix

In the NPC microenvironment, CD45^+^ immune cells usually outnumber tumor cells and CD45^-^ non-immune stromal cells. Indeed, fibroblasts represent a relatively minor but critical subpopulation that constructs a complex extracellular matrix (ECM). Fibroblasts are rarely infiltrated in non-malignant nasopharyngeal tissues, and hardly recruited in response to acute inflammation. Whereas in the TME, fibroblasts constitute approximately 2% of the total stromal infiltrates in the NPC microenvironment. The abundance of fibroblasts has also been validated *via* multiplex immunohistochemistry. A high density of α-SMA^+^ fibroblasts is found in 41.2% of primary NPC biopsies and 83.3% of metastatic NPC tissues. In two independent NPC cohorts, a higher density of α-SMA^+^ fibroblasts has been found correlated with shorter overall survival and lower 5-year survival rates in NPC patients, suggesting their utility as an independent prognostic factor (116, 117). Recruitment and accumulation of fibroblasts is a malignancy-dependent process found in varied cancers, since they can develop a tumor-promoting ECM and secrete varied cytokines that facilitate survival and metastasis. The function of ECM is primarily involved with the activation of focal adhesion-related pathways, including FAK/SRC, PI3K/AKT and RhoA/ROCK signaling. The predominant ECM components in the NPC microenvironment are collagen (especially type I collagen), lumican, and fibronectin, which all have a positive impact on angiogenesis and anti-apoptosis (50, 118, 119). Other collagens are also synthesized by intratumoral fibroblasts, such as type III and type IV collagens, but they exhibit patient-specific distribution. Recent advances have suggested that the tumor-parenchyma barrier manipulated by fibroblasts is the first protective shield of tumor cells to attenuate infiltration of T cells and penetration of anti-PD-1 drugs in patients with lung cancer and esophageal cancer (120). Hence, ECM is one of the critical mediators of immune suppression in the NPC microenvironment.

Besides, fibroblasts in the NPC microenvironment can secrete varied growth factors, including EGF, FGF, IGF1, CSF and TGF-β, which can either facilitate tumor progression or immune suppression (121). For example, CSF-1 is a vital factor inducing M0-to-M2 polarization, TGF-β induces differentiation and activation of Tregs, and IGF-1 is positively correlated to tumor sizes in NPC patients (122, 123). Fibroblast growth factor 2 (FGF2) is the upstream molecule of the PI3K/AKT signal pathway and activates proliferation and metastasis of NPC cells so that FGF2/FGFR2 has become a crucial target in the treatment of NPC as well (124). Targeting fibroblasts in the NPC microenvironment might provide significant benefits to chemo and immunotherapy. These fibroblasts can also secrete varied chemokines, such as CXCL9, CXCL10 and CXCL12 which can promote tumor growth and has chemoattractant properties that stimulate the migration of CXCR3^+^/CXCR4^+^ suppressive Tregs and cytotoxic T cells into the NPC microenvironment (24, 56). However, the fibroblasts can quickly inhibit the cytotoxic function of these recruited T cells *via* PD-L2-PD-1 interaction. Some fibroblasts also secret immunosuppressive factor IDO1, which further regulate T-cell immunity in the NPC microenvironment (24, 56). Targeting fibroblasts with high expression of fibroblast activation protein-α (FAP) in tumor-bearing mice have induced tumor necrosis mediated by IFN-γ and TNF-α, demonstrating that anti-tumor immunity has been reverted upon depletion of these cells (125). Significantly, up-regulated genes in tumor-derived fibroblasts were enriched in IFN response-related pathways (23). Meanwhile, fibroblasts are also considered as one of the vital factors maintaining the differentiation status of NPC cells because they are frequently associated with the epithelial-to-mesenchymal transition (13, 126). Targeting infiltrating fibroblasts might also result in decreasing the cancer stem cell pools in NPC to alleviate therapeutic resistance commonly possessed by high stemness cells (126). CD248 has been found dynamically expressed by cancer-associated fibroblasts (CAFs), and lowly expressed by other stromal cells (127). Although, it might serve as a potential molecular marker and target, the expression and mechanism of CD248 in NPC-infiltrating fibroblasts have not been fully elucidated, due to a low number of fibroblasts captured *via* single-cell sequencing. The current single-cell technique allows to process 20,000 cells per reaction, and it remains insufficient to depict the global mapping of stromal cells with low abundance in the NPC microenvironment. Thus, it is necessary to enrich the CD45^-^ non-immune stromal cells prior to sequencing so that we can have a maximum yield of fibroblasts to further identify the heterogeneity and molecular signatures of varied fibroblast subpopulations and characterize their functional status.

## Discussion

**Figure 2:**
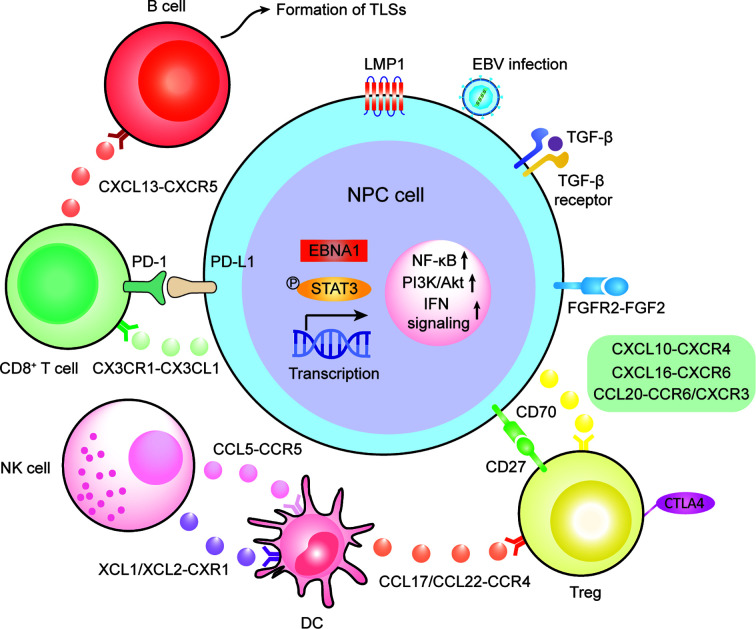
The schematic illustration of cell recruitment and cell-cell interactions in the NPC microenvironment.

The past two decades have witnessed a tremendous shift from recognizing the tumor as a homogenous entity towards understanding TME as a heterogeneous ecosystem at a single-cell resolution. Recent advances using single-cell sequencing and multiplex immunohistochemistry have deciphered the NPC microenvironment as a tumor-promoting and immuno-suppressive harbor. Single cell-cell communication analysis and V(D)J immune profiling has revealed the origin of tumor-infiltrating stromal cells so that we can know the recruitment mechanism and tumor-stroma interplay either from peripheral blood, adjacent lymph nodes and surroundings (**Figure 2** and **Table 1**). Nevertheless, we have seen a lack of functional and clinical investigations on the function and mechanism of T cells, NK cells, B cells, myeloid-derived cells and fibroblasts in the NPC microenvironment. Among these infiltrating stromal subtypes, B cells are the least studied subtype in the TME due to their relatively low infiltration degree in many malignancies. Therefore, we should continue to identify the finer B-cell subpopulations and characterize the functional status of B cell subtypes, since B cells are gradually recognized as a ineligible factor that influences the responsiveness of chemo and immunotherapy (128, 129). Besides stromal compositions in the NPC microenvironment, understanding the spatial orientation of stromal cells is also critical to functional dynamics and clinical translation. The field of spatial single-cell transcriptomics is rapidly evolving to uncover the locoregional localization of different stromal counterparts from formalin-fixed paraffin-embedded (FFPE) tissue samples (130). It is necessary to map where the cell-cell communication is occurring, but this technique highly relies on tissue quality and technical proficiency. So far, lack of reproducibility which induce high batch-to-batch effect and lower sequencing depth has hindered the feasibility of spatial single-cell transcriptomics. Single-cell sequencing is also currently facing limitations on sequencing depth in which lowly expressed genes cannot be detected even though they are highly important. Finer subpopulations, especially those that are lower than 5% of total cell input, cannot be accurately identified *via* sequencing nor characterized *via* computational algorithm. Thus, it remains necessary to enrich lowly infiltrated stromal cells prior to sequencing *via* flow cytometry and magnetic separation based on known signatures so that higher resolution of the molecular landscape can be reliably analyzed to depict the true molecular landscape and functional dynamics in the human body.

**Table 1:** Novel chemotaxis responsible for cell recruitment in the NPC microenvironment.

Chemotaxis (A-B)	Interacting cell subtype A	Interacting cell subtype B
CX3CL1-CX3CR1	Tumor cells	CD8^+^ T cells with minimal cytotoxicity
CXCL10-CXCR4	Tumor cells	Treg
CXCL16-CXCR6		
CCL20-CCR6/CXCR3		
CXCL13-CXCR5	Exhausted/helper T cells	B cells, except plasma B cells
XCL1/XCL2-CXR1	NK cells	Pro-inflammatory DCs
CCL5-CCR5		
CCL17/CCL22-CCR4	LAMP3+ DC	Treg
CCL19-CCR7		Naïve T cells
CCL4L2-CCR5	Macrophage	Memory T cells

**Figure 3:**
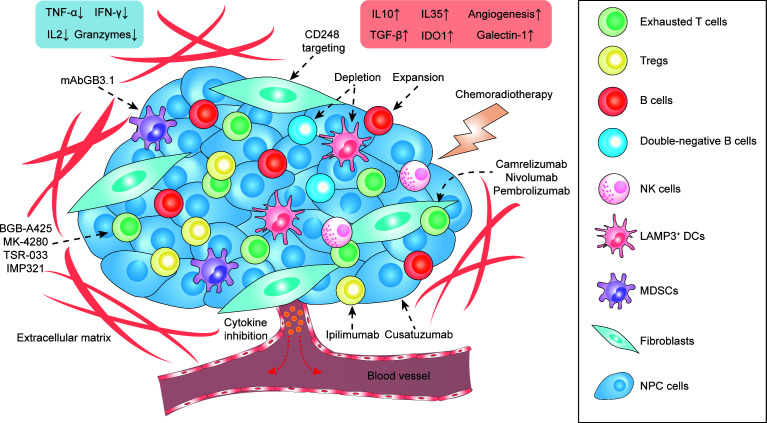
The stromal landscape of NPC at a glance and proposed treatment strategies targeting TME-infiltrating cells.

In this review, we have proposed a variety of therapeutic strategies that might enhance immunotherapeutic efficacy *via* direct and indirect remodeling of the NPC microenvironment (**Figure 3** and **Table 2**), which highly relies on recent identification and characterization of molecular targets that are essential for tumor proliferation and progression in NPC. Although some targets cannot be independently utilized as effective therapeutics like anti-PD-1/PD-L1 monotherapy, most of them remain feasible to be used as adjuvant therapies that enhance the efficacy of ongoing monotherapy in NPC patients. We acknowledge that there is a large transition gap between laboratorial investigation and clinical translation, but we believe that rational combinatorial strategies will be one of the most effective therapeutics in the future. However, functional analysis on NPC greatly suffers from the lack of reliable animal models. It is needed to establish a humanized mouse model with a competent human-originated immune system so that NPC-mediated immune regulation can be studied *in vivo* and predict clinical outcomes in NPC patients (131). Currently, NPC organoid is also underdeveloped in multiple groups around the world which can also serve an important alternative to study the function and interaction *in vivo* from NPC patient derived tissues, and to study the feasibility and efficacy of proposed therapeutic strategies (132). Due to the intrinsic nature of NPC as a stromal inflamed tumor, it would be better to adapt to local conditions to use tumor infiltrates themselves, instead of primarily focusing on extraneous attack mediated by chemotherapy and radiotherapy. Indeed, optimizing the innate and adaptive immunity to combat tumor progression is the principle of precision medicine and is currently leading the tide in the upcoming decade in cancer treatment. Better defining molecular contributors to an immunosuppressive and tumor-promoting microenvironment constitute an important step, including thorough investigations not only of locoregional stromal infiltration but also of such modifiable factors in peripheral blood, surrounding lymph nodes and metastatic sites. What lies beneath is a complex environment that supports the tumor, and we expect that targeting this foundation will yield the next breakthroughs in cancer therapy with greater efficacy, less toxicity, and less cost of cancer care. The development and use of such pharmaceutical agents targeting signature- and function-characterized populations enable a more personalized approach to NPC treatment.

**Table 2:** Proposed therapeutic approaches to target tumor-infiltrating cell subtypes in the NPC microenvironment.

Cell subtypes	Molecular targets	Outcomes	Available drugs or approaches
Tregs	CTLA4	Reduced T cell suppression	Ipilimumab
	CD27-CD70		Cusatuzumab
	LGALS1		N/A
Exhausted T cells	PD1-PDL1	Enhanced T cell cytotoxicity	Camrelizumab, Nivolumab, Pembrolizumab
	HAVCR2-LGALS9		BGB-A425
	LAG3		MK-4280, TSR-033, IMP321
CXCR5^+^ B cells	CXCL13-CXCR5	Enhanced responsiveness to immunotherapy	*Ex vivo* expansion
Double-negative B cells	NA	Better prognosis	Specific depletion/differentiation
DCs	LAMP3	Reduced T cell suppression	Specific depletion
MDSCs	S100A8/S100A9	Reduced chronic inflammation	mAbGB3.1
Fibroblasts	CD248	Inhibited recruitment of endothelial cells and angiogenesis	Targeted therapy

N/A, not applicable.

## Publisher’s Note

All claims expressed in this article are solely those of the authors and do not necessarily represent those of their affiliated organizations, or those of the publisher, the editors and the reviewers. Any product that may be evaluated in this article, or claim that may be made by its manufacturer, is not guaranteed or endorsed by the publisher.

## Author Contributions

X-YG and AW-ML supervised and reviewed the manuscript. LG conducted literature review and wrote the manuscript. DL-WK, WD, PW, and YW contributed to the clinical and bioinformatics interpretation of the manuscript. All authors contributed to the article and approved the submitted version.

## Funding

This work was supported by grants from the Hong Kong Research Grant Council (RGC) grants including GRF (17143716), Collaborative Research Funds (C7065-18GF and C7026-18GF), Theme-based Research Scheme (T12-704/16-R), National Key Sci-Tech Special Project of Infectious Diseases (2013ZX10002-011-005), and The Shenzhen Peacock team project (KQTD2015033117210153 and KQTD2018041118502879), X-YG is the Sophie YM Chan Professor in Cancer Research.

## Conflict of Interest

The authors declare that the research was conducted in the absence of any commercial or financial relationships that could be construed as a potential conflict of interest.

## Publisher’s Note

All claims expressed in this article are solely those of the authors and do not necessarily represent those of their affiliated organizations, or those of the publisher, the editors and the reviewers. Any product that may be evaluated in this article, or claim that may be made by its manufacturer, is not guaranteed or endorsed by the publisher.

## Abbreviations

TME, Tumor microenvironment; EBV, Epstein Barr virus; NPC, Nasopharyngeal carcinoma; TLS, Tertiary lymphoid structure; H&E staining, Hematoxylin and eosin staining; IHC staining, Immunohistological staining; NK cell, Natural killer cell; Treg cell, Regulatory T cell; IFN, Interferon; EBNA1, Epstein-Barr nuclear antigen 1; LMP1, Latent membrane protein 1; LMP2, Latent membrane protein 2; EBER, Epstein-Barr encoding region; MDSCs, Myeloid-derived suppressor cells; TCR, T-cell receptor; HNSCC, Head and neck squamous cell carcinoma; HPV, Human papillomavirus; GZM, Granzyme; DC, Dendritic cell; HCC, Hepatocellular carcinoma; NSCLC, Non-small-cell-lung cancer; ESCC, Esophageal squamous cell carcinoma; ECM, Extracellular matrix; FGF2, Fibroblast growth factor 2; FAP, Fibroblast activation protein-α; CAFs, Cancer-associated fibroblasts; FFPE tissue sample, Formalin-fixed paraffin-embedded tissue sample.
